# Noise in Schools: A Holistic Approach to the Issue

**DOI:** 10.3390/ijerph7083255

**Published:** 2010-08-23

**Authors:** Pamela Woolner, Elaine Hall

**Affiliations:** Research Centre for Learning and Teaching, King George VI Building, Queen Victoria Road, Newcastle University, Newcastle, NE1 7RU, UK; E-Mail: elaine.hall@ncl.ac.uk

**Keywords:** noise, learning environments, impact on students, participatory approaches

## Abstract

Much of the research evidence relating to the physical learning environment of schools is inconclusive, contradictory or incomplete. Nevertheless, within this confusing area, research from a number of disciplines, using a range of methodologies, points to the negative impact of noise on students’ learning. In this paper, drawing on our systematic review of learning environments we review the weight of evidence in relation to noise, considering what implications the results of these studies have for the design and use of learning spaces in schools. We make four key points. Firstly that noise over a given level does appear to have a negative impact on learning. Secondly that beneath these levels noise may or may not be problematic, depending on the social, cultural and pedagogical expectations of the students and teachers. Thirdly we argue that when noise is deemed to be a difficulty, this finding cannot simply be translated into design prescriptions. The reasons for this indeterminacy include differing understandings of the routes through which noise produces learning deficits, as well as relationships between noise and other elements of the environment, particularly the impacts of physical solutions to noise problems. Finally, we suggest that solutions to noise problems will not be produced by viewing noise in isolation, or even as part of the physical environment, but through participatory approaches to understanding and adapting the structure, organisation and use of learning spaces in schools.

## 1. Introduction: Investigating and Improving the Learning Environment

The learning environment provided by a school should be understood as resulting from a complex, dynamic relationship between the various physical elements and the attitudes and actions of the different users who constitute the school community. Therefore, although the identification of problems with the physical setting may be aided by a narrow focus, any attempts to improve the environment and facilitate better learning will require a wider perspective.

This reasoning underpinned our systematic review of research evidence relating to the physical learning environment [1] and led us to consider the evidence for impacts on learners’ attainment, engagement, affect, attendance and wellbeing. Table 1 summarises our findings in these terms, but also conveys our conclusion that considerably more evidence exists for the detrimental effects of poor environments than for the benefits of better environments. That is to say, there is a great deal of very convincing evidence that in a school built next to a motorway or an airport, there will be discernable impacts on learners’ comprehension, language learning, attention and other cognitive functions [2,3]. However, there is not the same calibre of evidence to suggest that there are predictable effects when noise in classrooms fluctuates in the middle ranges, for example to suggest that the noise made by learners engaged in active or collaborative activities has a negative impact on the amount or quality of what is being learned. As can also be seen in table 1, there is a range of research evidence relating to noise, which we will now consider in more detail.

## 2. The Nature of the Research Evidence

Understanding the relationship between the physical school environment and the educational activities that take place there is not a new problem. Potentially relevant information comes from a wide range of sources including current experiences and historical examples [5,6], as well as more systematically collected data. This research evidence is reported by studies originating in a diverse array of disciplines including experimental, environmental and health psychology, education, design and architecture, building management and public health. The resulting variety in methodological assumptions, research designs and preferred methods lead to difficulties in establishing an overview about levels of impact of the physical setting on students’ wellbeing, attitudes and learning [4]. It must be noted, however, that they also suggest a potential for the triangulation of evidence, together with providing a generally broader and more nuanced understanding of the complex relationship between setting and learning, both during daily use and when changes are made.

Within the variety of research there is a subsection which considers elements of the built environment in relative isolation. These studies tend to assess the impact of different levels of a quantifiable aspect, such as noise, daylight or air pollutants on measurable outcomes relating to health, psychological functioning or learning. Yet even such apparently well-controlled research still produces results which are often inconclusive and sometimes contradictory [see 4, p. 56].

## 3. Evidence about the Impact of Noise

### 3.1. Focused Studies

Much of the quite extensive research evidence relating to the issue of noise in education has been produced by studies of the sort mentioned above, focussed on relating noise levels to particular outcomes. Notably, the results, using both experimental and observational methodologies, are remarkably consistent. The findings show that noisy conditions have direct negative effects on learning, particularly language and reading development, as well as causing indirect problems to learners through distracting or annoying them. Laboratory based cognitive psychology experiments have shown that noise affects performance on memory tasks [7,8], an effect which is at least partly explained by noise interfering with language processing. This suggests that it might be problematic to live in a generally noisy place, and real world research into the effects of chronic noise, experienced by people living near airports or busy roads, confirms this extrapolation [9]. A review of this area concluded that, ‘The evidence for effects of environmental noise on health is strongest for annoyance, sleep and cognitive performance in adults and children.’ [10, p. 253, see also 11].

Of particular concern for education is the reliable finding, which fits with the laboratory results, that chronic noise exposure impairs cognitive functioning [see 12 for an overview]. Studies have found associations between noisy environments and reading problems [13–15], deficiencies in pre-reading skills [16] and more general cognitive deficits [17]. There is the implication that in addition to interfering with processing on each occasion, the environmental noise may be contributing to developmental problems, particularly with speech and language and with reading. These studies imply that either living in a home or attending a school near a source of on-going noise will increase the likelihood of a child having educational difficulties. Clearly those attending neighbourhood schools are likely to experience raised noise levels from the same source at both home and school. Shield and Dockrell make this point in their review of research concerning noise experienced by school students [12, p. 102], also noting that many such children suffer other problems of deprivation, presumably because noisy residential areas are not desirable places to live.

Recent research, however, into the problem of noise in the school environment has tended to centre on the potentially more widespread problem of students struggling to learn because of noise generated within the school itself. In their surveys of external and internal noise in urban primary schools, Shield and Dockrell comment that ‘during lessons it was normally not possible to hear external noises’ [18, p. 734] and the noise levels they report just outside these urban schools were frequently lower than might have been expected due to schools being located in side streets or separated from roads by playgrounds.

Possibly problematic noise comes from several sources, as demonstrated by studies where measurements of classroom noise levels were made [18–21]. Firstly, there is noise intrusion, which can come from outside the school, but seems to be increased more by noise from other parts of the school leaking into the classroom. Secondly, there is background noise from within the room, often due to heating and ventilation systems but also caused by equipment such as projectors and computers. Finally there is the noise generated by students engaged in learning, which, unsurprisingly, varies according to the nature of the activity [18, p. 735]. Noise levels are argued to be exacerbated by high levels of reverberation, since this can increase the noise level itself and make the hearing of speech more difficult [20, p. 7]. The consistent findings, across laboratory and field studies, of noise interfering with language based tasks together with concerns about speech intelligibility in the classroom has suggested to some that controlling reverberation might be as important as reducing noise levels. Indeed this is an argument that some researchers advance [19,20], although other work is more equivocal about reverberation, tending instead to emphasise noise levels and signal to noise ratios in discussions of speech intelligibility [12], standards for school building adopted or advocated always include guideline maximums for both noise levels and reverberation [12,22–24].

Potentially linking both external and classroom noise directly with cognitive processing deficits, there have been some experimental studies which have investigated how well children perform various learning tasks when exposed to recorded real world noises played at a range of levels. Dockrell and Shield [25] used recordings of classroom ‘babble’ alone and together with environmental noises such as sirens and road noise. These noises were played at a level to match average ambient noise levels recorded in classrooms, and tended to impair performance on a range of literacy, numeracy and speed tests. There was, however, complexity in the results relating to the impacts of the differing sorts of noise on the processes underlying performance on the various tests.

Hygge [26] conducted a series of experiments where school children read texts in either noisy or quiet conditions, with the noise provided by recordings of aircraft, train and road noise or irrelevant speech. Tests conducted a week later in quiet conditions showed deficits in memory for these texts when they were studied in noisy conditions, with this being more pronounced for recall, as opposed to recognition, of information. Interestingly, this deficiency in learning continued from session to session when children had experienced noise first, associated with motivation for the task dropping off as a result of the noise experience.

Thus, these sorts of experiments demonstrate not only that children generally have more difficulty performing cognitive tasks when it is noisy, but also suggest that noise tends to undermine long term learning, corroborating the findings of the observational studies of chronic environmental noise. Yet, in both the experimental studies discussed above there were complications in the patterns of results which leave uncertainties about precisely how noise interferes with particular cognitive processes relevant to learning.

### 3.2. Wider Frames of Noise Research

Other research has been conducted that takes a somewhat wider view of noise, sometimes also attempting to understand the mechanisms for the effects reviewed above. As Hygge and Knez point out, there is “theoretical and practical value in knowing how the physical parameters of the indoor environment may combine or interact” [27, p. 291], but very little work puts this into practice. Their own studies of noise in combination with heat and lighting [27,28] are an exception to this, but have failed to clarify when and to what extent problems due to these other physical factors interact with deficiencies caused by noise.

An important interaction with absolute noise levels are the attempts that teachers and learners make to accommodate or compensate for noisy surroundings. Early research observed that teachers pausing during bursts of external noise leads effectively to a reduction in teaching time, sometimes estimating this loss to be as high as 11% of teaching time [15]. More recently, researchers in the US have found evidence of voice fatigue being a particular health concern for teachers [29], and this is identified as another reason to reduce classroom noise levels [20, p.3,23]. Turning now to the actions of the learners in noisy situations, it has been suggested that apparently conflicting experimental results may sometimes be due to the positive effect of arousal [27]. In discussing the results of their noise experiment, Dockrell and Shield argue that children in the environmental noise condition may have been provoked ‘to actively focus on the task, possibly by redirecting attention’ [25, p. 522], with this heightened attention producing better than expected performance. From these experimental studies, however, it is not possible to know the limits of such improved performance over time, different tasks and noises, or across differing learners.

Much work does not differentiate between individuals in their reactions to noise or between different sorts of noises. Where individual differences are investigated, there is a body of evidence that some individuals might be more sensitive to noise than others [30,31]. The more immediate problem for school design in terms of inclusion is the finding from some studies that the performance of students with Special Educational Needs (SEN) might be more susceptible to noise [25], although impacts on particular children may not be predictable [32,33]. Also, learners for whom the language spoken at school is an additional, rather than first, language may be further disadvantaged by noise in the classroom [11]. Nelson *et al.* [20] review research evidence showing that such children perform less well than native speakers on tests of speech comprehension against noise. On the written tasks used by Dockrell and Shield [25], however, the performance pattern of the children with English as an additional language (EAL) did not suggest that they suffered additional difficulties, probably because the tasks did not depend on speech intelligibility. Considering different types of noise, Hygge [26] presents evidence of aircraft noise being particularly disruptive to learning, traffic noise less disruptive but still impairing performance, while train noise did not cause a problem. Yet, confusingly, this study also found no effect for verbal noise, despite Knez and Hygge [27] arguing that irrelevant speech is a particularly distracting noise.

These apparent contradictions may be explained by research findings that factors such as predictability, control and judged necessity influence how annoying people find particular noises [10,34], although the relationship of noise annoyance and performance deficits is far from clear. There is interest in noise annoyance [34,35] and links to mood [36,37], but Hygge [26] argued that interference with the encoding stage of memory which the noise conditions seemed to cause in his experiment was not mediated by distraction or mood. Related to this, research by Dockrell and Shield [38] into the perceptions of primary school children regarding actual environmental noise showed that annoyance may not be related in a straightforward manner to either noise level or frequency of occurrence.

Within mainstream education literature there appears to be more awareness of noise generated within schools, rather than externally, and the impact is usually considered through investigations of perceptions of this noise and any annoyance or sense of distraction. For example, Flutter has spent more than a decade involved in projects where students are consulted about their learning and has found that a central concern is with excessive noise: ‘Noise and distracting behaviour were the most frequently mentioned problems and many students said they would like a calmer and quieter environment’ [39, p. 184]. In answer to this, a book about effective teaching argues that a whole school policy addressing corridor noise levels has the potential to improve ‘classroom climate’, making it more conducive for learning [40, p. 61]. Recent rebuilding and refurbishment programmes within the UK [41,42] have also alerted some educators to the contribution of qualities of the school building itself to experiences of noise. In English secondary schools awaiting Building Schools for the Future (BSF) rebuilding work, problems with acoustics are considered by over three-quarters of head teachers to be affecting to ‘a large extent’ or ‘to some extent’ the school’s provision of education [41, p. 65]. Such reports of inadequate acoustics are likely to be broadly accurate since these schools will have been constructed according to less stringent guidelines, which were not then legally compelling [see 12, pp. 108–109]. Furthermore, many of these schools were built in the second half of the twentieth century using lightweight construction methods with inadequate sound insulation. Since similar designs and construction methods extend across the sectors of education and beyond the UK, this is not a problem specific to UK secondary schools although the BSF programme has highlighted it here.

### 3.3. Lessons from the Research Evidence

As stated at the beginning of this section, the research evidence relating to noise is remarkable for its broad consistencies. Noise interferes with learning, through direct effects on information processing, and via indirect effects on teachers, learners and communication in the classroom. The variety of these routes suggest why noise is a serious problem for schools but also why the causal mechanisms proposed, principally interference with speech intelligibility, distraction and annoyance are each unlikely to provide complete explanations of how noise interferes with learning. It is clear that high background noise levels and poor acoustics decrease speech intelligibility but educational activities are not all equally dependent on understanding speech, as will be discussed in more detail below. Although noises may (sometimes) annoy people, or cause distractions that could impair learning, neither of these causal links seems completely necessary for problems with learning, and are likely to be only part of the problem, or perhaps are only significant in some cases.

### 3.4. Solutions to Noise Problems in Schools

The range of noise sources and the various ways in which noise can impair learning, which the research base, and the differing interpretations of its findings, illuminate are indicative of a serious problem for schools. Yet the nature of this research also suggests the difficulty of enacting solutions to these noise problems. Firstly, it is clear that different solutions will be required depending on the main source of the noise. Concerns about external environmental noises affecting health, wellbeing and learning in school have implications for the location and design of the school structure. This issue is discussed, for example, in the World Health Organisation (WHO) guidelines, where recommendations for planning bodies and architects are laid out [23, p. 10, p. 19]. Noise leaking between rooms in a school can similarly be understood as a problem of the design and construction of the premises, while background noise due to heating and ventilation machinery suggests a need for quieter models [20, p. 8]. Across these noise sources, however, the emphasis of the solutions will depend on judgments made about the main mechanism for interference with learning. This is shown in relation to the proposal that teachers’ voices should be amplified [43]. This makes sense if the impact of the signal to noise ratio on speech intelligibility is considered central, but may be disputed if absolute noise levels are argued to be more important [20, p. 3].

Of distinct importance in identifying solutions to noise problems is the recognition that in educational settings, as in other real world situations, noise is not the isolated factor which it sometimes appears to be in the context of experimental and observational studies focused on noise. This means that proposed solutions for improving acoustic elements of the school environment might have detrimental effects on other aspects, while changes made to the physical setting to enhance other factors might worsen noise problems. A particular tension is evident between noise and air quality. Some suggested acoustics improvements, such as carpets [21] or ceiling hangings [16] may collect dust and so worsen air quality, while excessive background noise in classrooms is often due to open windows [23, p. 8] or mechanical ventilation units [19]. Yet research demonstrating the impact of poor air quality on health, absence from school [44], and therefore on learning, suggests that good ventilation is vital in a classroom, a conclusion that reviews of the physical education environment tend to reach [45–49].

Other interactions of noise with various environmental factors, and the potential for solutions to conflict, are evident from considering studies in these separately researched areas. User satisfaction with buildings has been found to be linked to the control of temperature [50, p. 133], but this again suggests teachers opening windows and turning on heaters, with the associated implications for classroom noise levels. There is also research suggesting that more spacious classrooms result in more positive feelings amongst students [51] and, very specifically, that higher ceilings in classrooms decrease perceptions of crowding, and increase teacher satisfaction [52]. Yet larger spaces present more acoustic challenges, particularly in relation to reverberation. Measurements reported by Knecht et al showed that ‘rooms with the longest reverberation times were those with the largest volumes’, and the classrooms with lower ceilings were more likely to have ‘acceptable’ reverberation times [19, p. 69]. The inevitable difficulties of resolving these tensions and interactions satisfactorily are very rarely suggested by the building guidelines relating to noise. A notable exception to this omission is the discussion in the WHO pamphlet about the need for school canteens to be both hygienic and acoustically satisfactory, yet ‘the materials available rarely meet the two criteria simultaneously’ [23, p. 15]. The authors then go on to make practical suggestions, such as the use of tablecloths and rubber pads on chair legs, which will reduce noise levels without compromising hygiene.

Adding another level of complexity to the competing demands of the physical environment is the fact that inhabitants of a setting are not passive recipients, and their actions will interact with aspects of the surroundings, sometimes in unpredictable or complicated ways. This has already been suggested by descriptions above of teachers pausing during occasions of external noise and suggestions from some noise experiments that potentially distracting noise may sometimes enhance performance as people make more effort to concentrate. Other instances of changed behaviour due to noise might be more predictable, but are no less problematic. The WHO guidelines describe the ‘vicious circle’ that occurs when inadequate acoustics force speakers to raise their voices, resulting in more noise and so yet louder voices [23, p. 9].

These examples indicate the potential for more extended modifications to behaviour to have pronounced effects on the learning environment, and indeed there is some research evidence of this occurring. In the 1960s and 1970s when many new schools in the US and UK were built around open plan principles, some investigations of teaching practices in these new settings found that teachers were being more, rather than less, conservative in their methods. Fears of disturbing other classes made them less likely to encourage active, and so more noisy, independent learning than were teachers in traditional cellular classrooms [52,53]. Similarly, some research into behaviour in open plan offices shows such settings can discourage, rather than facilitate, social interactions, as people are concerned about disturbing their colleagues [54]. These observations suggest that sometimes in schools an organisational approach to noise nuisance may be appropriate so that a peaceful working environment can be maintained without the loss of other school activities that generate more noise. One of the WHO recommendations asserts that schools ought to ‘adapt their timetables in order to set aside certain periods of the day and areas of the school for noisy activities’ [23, p. 19].

It is worth remembering, however, in connection with organising and managing a school community to control noise that such institutional management necessitates value judgments being made about different sorts of noise. Also, in this context, it is worth noting the considerable disagreement within the noise literature about the importance of conscious noise annoyance and the range of factors which appear to affect individual judgments [e.g., see 38, p. 2965]. Although these judgments, both those made by individuals and those reached at an institutional level, are undoubtedly more true to the complexity of human experience of noise than purely quantitative measurements of noise level, they do leave more room for argument. For example, in a recent paper a British education researcher argues that existing structures in schools result in ‘weak silences’, consisting of imposed quiet which disempowers students through silencing them, and an absence of ‘strong silences’, when non-coercive quiet allows concentration, gaze and self-understanding [55]. This suggestion that there exist bad sorts of quiet in school is balanced by understandings, common among educationalists, that there may be good noise. The classroom activities which Shield and Dockrell found to be noisiest [18, p. 735], ‘group work’ and ‘group work and movement’, are considered by many educators to be desirable, through facilitating learners’ engagement and collaboration. Furthermore, theorists about educational practices often argue for the centrality of dialogue, both between teacher and learner and between learners, to successful learning [56,57].

## 4. Conclusions

It is clear that a range of approaches to noise problems in school are required. Appropriate solutions will depend partly on the nature of the noise, with differing solutions being needed for different sorts of noise. The impact of external noise on health and happiness, as well on learning, has distinct implications for the planning and architecture of schools (as acknowledged for example by the WHO guidelines). Noise leaking between rooms suggests improved sound insulation while high levels of background noise in classrooms indicate a need for quieter models of heating and other equipment. Yet it is also clear that solutions to noise in school will differ according to differing emphases on causal factors and differing understandings of the nature of the problem.

Models of noise nuisance which centre on noise impacting on cognitive processes, such as attention or language, and those which prioritise noise annoyance or impacts on mood, may suggest different mechanisms but they have in common an individualised view of learning and, indeed, existing. A more collaborative perspective is evident in noise research and related building guidelines that focus on speech intelligibility. In the context of learning in school, this seems a more appropriate and realistic outlook, but also appears to rely on a transmission model of learning which many educationalists and developmental psychologists would consider simplistic and outdated. This viewpoint is evident, for example, in the WHO’s stated assumption that ‘In schools, teaching is based to a very large extent on oral communication’ [23, p. 5], a view which assumes that the communication in question is one voice to many ears, while in their review of noise in school, Shield and Dockrell claim that ‘the major function of a classroom [lies] in providing an environment that enables the transfer of information from teacher to pupil’ [12, p. 99], missing the inter-personal and co-constructed nature of a great deal of learning in schools. It seems clear that these understandings of noise in relation to learning only relate to partial understandings of the diverse and rich classrooms and the multitude of learning opportunities in education. Too complete a concentration on this view of noise would surely produce oppressive hush in schools and a narrowing of the educational opportunities offered. If the importance of dialogue is as high as is claimed by some educators, such a response to noise could even end up being as detrimental to learning as chronic external noise is known to be.

## A Participatory Approach to Noise in School

The research base suggests that quantitative measurement of noise levels allied with physical design solutions may be appropriate for the solution of big, primarily external, noise problems, such as those due to roads, flight paths and, perhaps, noisy school equipment. Once such problems are solved, or if they are not actually problems for a particular school, however, the recurring difficulty for a school community is knowing how to understand and relate to noise more generally. This will be important for the schools where problems with acoustics are acknowledged (as in many of the UK schools awaiting BSF rebuilding work), but the research findings of noise directly impacting on some cognitive processes also indicates that consideration should be given even in schools where noise is not recognised as a particular problem. However, the understanding developed in this article makes clear that investigations into noise need to go beyond the measurement of noise levels. We would urge that high levels of noise be addressed to protect learners from damage but, in the mid-range, we counsel for a development from a purely quantitative idea of acceptable/unacceptable levels of noise towards a more nuanced understanding of the context in which noise is generated and the kinds of learning activity taking place. Although measuring noise levels might form part of preliminary work and the published guidelines may be useful in interpreting the findings, the wider arguments of some of these publications (the WHO document is particularly good in this respect) need to be considered and understood in the context of the culture and values of the educational system and of the particular school. The centrality of individual and institutional judgments, both to developing understanding of noise in a particular setting and to the sorts of solutions which will be appropriate to any problems, implies that a participatory approach taken at the school level will be most successful.

We have previously developed arguments for the necessity of participatory approaches to altering or improving the learning environment provided by a school [58–60]. However, it is useful to consider how current knowledge of participatory practices might apply specifically to noise in school. While remaining mindful of the fact that participatory approaches can add complexity to processes, so participation should not be viewed as a panacea, it is possible to identify three broad indicators of success. These three indicators were identified in relation to participatory approaches to changing the social and physical experience of break time in school [61], but would seem to apply much more generally to any attempt to understand, and so improve, the ongoing relationship that school users have with their physical environment. These authors argue firstly for the need for a holistic approach, considering all aspects of the break time situation, including the physical space, together with the management and organisation of time, space and people. Any change must recognise and encompass all these aspects; otherwise it will be short-lived and superficial. Similarly, it has been argued here in the context of noise that the quantifiable side to noise problems and their solutions cannot be seen in isolation from wider understandings of the physical environment together with shared conceptions of learning and education.

The second conclusion about break times, and more generally about collaborative change in school, is that for attempts to succeed they must involve the whole school community. The authors note that the phase ‘whole school approach’ is over-used but they emphasise that there is a central ‘need to involve all in a meaningful dialogue about change’ [61, p. 190]. Within BSF in the UK, facilitator John Mitchell has recently concluded from his experiences that one of the main requirements for success is ‘whole school involvement’ [62, pp. 244–245]. A whole school approach to noise seems additionally appropriate given the advice of the WHO to use organisation and management, such as timetabling and zoning within the premises, to address problems with noise. In contrast, an example of the disadvantages of schools taking a more fragmented approach can be seen in a study of classroom noise which discovered an excessively noisy fish tank [19, p. 68], which would perhaps be more appropriately housed in the school hall or entrance area.

Finally, in connection with making changes to school break times, it was concluded that it is important to understand that any process of change is not straightforward. Similar conclusions can be drawn from a recent international review of whole school change [63]. In the context of the physical school environment, and noise in particular, it seems necessary for all those involved to acknowledge this. As has been argued above, the consistent findings of noise causing problems in learning do not produce prescriptions for the design or use of an educational setting. Any genuinely collaborative approach will not be simple to enact given the importance of establishing shared conceptions of learning and education before problems can be understood and solutions can be tried.

These three broad themes to successful negotiation of change in the school environment all show the necessity of genuine collaboration, as well as providing some suggestion of how to succeed. Evidence and experience, as reviewed in this paper, indicates the appropriateness of such an approach in the case of noise in schools and see also [64–66] for more international perspectives. The more detailed consideration of these aspects of successful participatory approaches in relation to noise, which was briefly suggested above, further implies the usefulness of this framework for schools addressing concerns about noise. It can be seen that, as suggested in this paper’s introduction, a narrow view of physical aspects of the learning environment may be sufficient to demonstrate the negative effects of noise on learning. Yet a wider perspective will be necessary to achieve solutions to learning problems due to noise which avoid producing detrimental effects on health and wellbeing, as well as on some of the wide range of actions that comprise learning. It seems likely that understanding noise in relation to other physical variables at both the classroom and school level, together with developing a shared overview of learning, will be best achieved through a participatory approach. This might start from measuring noise levels and comparing with published guidelines, but clearly needs to develop beyond this.

## Figures and Tables

**Table 1.** Summary of evidence of effects (adapted from [4]).

	Types of evidence relating to impact on learning				
	Attainment	Engagement	Affect	Attendance	Wellbeing
Poor quality environment has detrimental effect	Noise Air quality Safe, healthy surroundings	Noise Air quality	Noise Build quality	Air quality Safe, healthy surroundings	Air quality Lighting ICT
Equivocal evidence	Room arrangement	Noise (learned helplessness) Ergonomic furniture Temperature Air Quality Room arrangement	Noise (mood) Colour Lighting Build quality	Temperature Air quality Build quality Lighting	Ergonomic furniture
Adding value to adequate environment has benefits	Daylight Build quality	Ceiling height Colour Storage	Beautiful spaces Ceiling height Display		
